# Towards a patient-based drug discovery

**DOI:** 10.1186/1753-6561-6-S6-O30

**Published:** 2012-10-01

**Authors:** Stuart L Schreiber

**Affiliations:** 1 Broad Institute of Harvard and MIT, MA, USA

## 

Small-molecule drugs were originally discovered using compound-based drug discovery: opportunistic discovery of a biologically active compound, often a natural product (such as penicillin) followed by a search for a disease that might be treated with the compound. This remains a common approach to modern drug discovery (for example, rapamycin and analogs for use as antifungal agents, immune suppression agents, anti-cancer agents, and possibly others in the future).

The advent of recombinant DNA accelerated a second approach -target-based drug discovery - where the therapeutic target is selected and subjected to methods that yield candidate drugs (mechanism-based design, structure-based design, screening). But this approach has its shortcomings - 97% of drug candidates that enter into clinical investigation eventually fail, many due to unanticipated toxicity and many others due to a lack of efficacy despite successful modulation of the target. Selecting therapeutic targets based on information derived from surrogates of patients has proved challenging.

Advances in human biology, including human genetics and physiology, and in small-molecule science, including chemistry and chemical biology, are now accelerating a third approach: patient-based drug discovery. I will present examples that aim to use: 1) information from heritable or somatic human genetics in human disease, for example, in Crohn's disease and cancer, 2) advances in diversity-oriented synthetic chemistry and chemical biology to accelerate the discovery of safe and effective small-molecule therapeutics, and 3) an understanding of the relationship of human genetic variation with drug efficacy.

